# Psychosocial Mechanisms of Successful Aging Among Older Women Living With HIV: A Structural Equation Model

**DOI:** 10.1093/geroni/igaa057.1613

**Published:** 2020-12-16

**Authors:** Anna Rubtsova, Gina Wingood, Igho Ofotokun, Deborah Gustafson, David Vance, Anjali Sharma, Adaora Adimora, Marcia Holstad

**Affiliations:** 1 Emory University, Atlanta, Georgia, United States; 2 Columbia University Mailman School of Public Health, New York, New York, United States; 3 Emory University School of Medicine, Atlanta, Georgia, United States; 4 SUNY Downstate Medical Center, Brooklyn, New York, United States; 5 The University of Alabama at Birmingham, Birmingham, Alabama, United States; 6 Albert Einstein College of Medicine, Bronx, New York, United States; 7 University of North Carolina at Chapel Hill School of Medicine, Chapel Hill, North Carolina, United States

## Abstract

Although older women living with HIV (OWLH) face challenges related to the intersection of HIV and aging, our published research found significant prevalence of self-rated successful aging (SRSA) in a sample of OWLH enrolled in the Women’s Interagency HIV Study (WIHS). Studies in other populations link SRSA to positive psychosocial factors but little is known about SRSA mechanisms among OWLH. The purpose of this study is to test a conceptual psychosocial model of SRSA. Our sample (N=356) included OWLH enrolled in WIHS who participated in the “From Surviving to Thriving” (FROST) substudy and completed psychosocial and cognitive assessments: average age 56.5 years, 73% Black, 55% with annual income ≤ $12,000, 74% having 3 or more comorbidities, median CD4=673 cells/ml (Q1=486; Q3=880). SRSA was assessed using a research-based 10-point scale (higher scores=better outcomes). We conducted adjusted structural equation modeling. The global model included two latent variables -- protective attributes (composite of positive psychosocial factors: resilience, personal mastery, optimism, spirituality) and negative affect (composite of negative psychosocial factors: anxiety, depression, loneliness, internalized HIV-related stigma). The model showed good fit (χ2(65)=72.3, p=0.25; RMSE=0.02; CFI=0.99) and explained 21% of variance in SRSA. Increased protective attributes were associated with improved SRSA both directly (p<0.01) and indirectly, via improved coping with stress (p<0.001). While negative affect did not have a direct effect on SRSA, it was indirectly associated with worsened SRSA via diminished protective attributes (p<0.001). Findings suggest the need for interventions enhancing positive and mitigating negative psychosocial factors to promote SRSA among OWLH.

